# Changes in VO_2_max and cardiac output in response to short-term high-intensity interval training in Caucasian and Hispanic young women: A pilot study

**DOI:** 10.1371/journal.pone.0244850

**Published:** 2021-01-22

**Authors:** Jamie L. De Revere, Rasmus D. Clausen, Todd A. Astorino

**Affiliations:** Department of Kinesiology, California State University—San Marcos, San Marcos, California, United States of America; Pennington Biomedical Research Center, UNITED STATES

## Abstract

Data obtained in primarily Caucasian (C) and African American adults show that ethnicity does not mediate responsiveness to exercise training. It is unknown if Hispanics (H), who face elevated health risks and are less active than C, exhibit a similar response to exercise training. This study compared cardiorespiratory and hemodynamic responses to high intensity interval training (HIIT) between C and H women. Twelve C and ten H women ages 19–35 yr who were non-obese and inactive completed nine sessions of HIIT over a 3 wk period. Maximal oxygen uptake (VO_2_max) was assessed twice at baseline during which thoracic impedance was used to evaluate heart rate (HR), stroke volume (SV) and cardiac output (CO). Habitual physical activity was assessed using accelerometry. Results showed a significant main effect of training for VO_2_max in C and H (F = 13.97, p = 0.001) and no group by training interaction (p = 0.65). There was a main effect of training for CO and SV in C and H (F = 7.57, p = 0.01; F = 7.16, p = 0.02), yet post hoc analyses revealed significant increases were only exhibited in C. There was a tendency for a group by training interaction for a-VO_2_diff (F = 1.32, p = 0.054), and a large effect size was seen in H (d = 1.02). Overall, data show no effect of ethnicity on changes in VO_2_max with low-volume HIIT, yet C and H may achieve this outcome differently. Longer studies in similar populations are needed to verify this result.

## Introduction

About 25% of adults meet the current Physical Activity Guidelines which suggest a minimum of 150 minutes of moderate intensity continuous training (MICT), 75 minutes of vigorous exercise, or a combination of both to achieve various health benefits [1]. Lack of physical activity increases risk of heart disease, stroke, type-2 diabetes, and certain cancers which increase morbidity and all-cause mortality [2]. Despite these well-documented benefits of MICT, many individuals express a perceived lack of time as a primary barrier to regular physical activity [3].

An alternative to MICT is high intensity interval training (HIIT), defined as repeated, brief, and intense exercise bouts separated by active recovery. It is apparent that HIIT elicits significant increases in VO_2_max [4] and fat oxidation [5], in turn improving exercise capacity and reducing health risks. Compared to MICT, increases in VO_2_max are frequently superior [6, 7] in response to HIIT despite the lower training volume. In addition, HIIT is equally or more enjoyable than MICT [8, 9] which suggests that it is a practical and tolerable exercise mode for many individuals including inactive adults. Although enjoyment is a multifactorial outcome, this characteristic of HIIT is critical to broaden its application to the general population, as exercise adherence is related to enjoyment [10].

Despite the widely documented benefits of HIIT, it is evident that not everyone responds equally to interval training or MICT. Data from inactive adults who underwent 20 weeks of MICT in the HERITAGE study [11] showed a mean increase in VO_2_max equal to 400 mL/min, although the magnitude of increase ranged from little to no response in some individuals to greater than 1 L/min in others. There is also individual responsiveness to HIIT and its more intense form, sprint interval training [12, 13]. The incidence of individual responses to training is important considering the relationship between increases in VO_2_max induced through physical activity and morbidity and mortality. For example, Kodama et al. [14] reported that every 1 MET increase in VO_2_max was associated with increased survivability of coronary heart disease (CHD) and cardiovascular disease (CVD) in adults by 13 and 15%, respectively. Data [11] reported that approximately 50% of the training-induced increase in VO_2_max is genetic, with the other 50% likely due to factors including habitual physical activity, dietary patterns, sleep quality, and characteristics of the physical activity regimen [15].

One widely ignored aspect of the individual response to exercise is ethnicity. Prior studies investigating the effect of physical activity on indices of cardio-metabolic health and mortality risk were performed in Caucasian (C) and a lesser amount of African American (AA) individuals including HERITAGE [11], STRRIDE [16], and DREW [17]. Their data suggest no difference in the adaptive response to MICT between C and AA. More recent data also suggest that changes in health-related outcomes across various ethnicities are similar. In a comparative cohort analysis examining “at-risk” African American and Caucasian women’s responses to exercise training, Bowdon et al. [18] concluded that both cohorts exhibited significant and similar reductions in blood pressure, exercise heart rate, and blood lipids. Conversely, Johnson et al. [19] displayed a different training-mediated change in total cholesterol, intermediate-density lipoprotein, triglyceride, and high-density lipoprotein size between C and AA. This suggests that ethnicity may affect the physiological adaptation to exercise and merits further study to investigate the effect of ethnicity on exercise responses in other populations.

Hispanic individuals (H) comprise an ever-increasing number of adults in the United States [20], as since 2000, the Hispanic population has expanded from 35.7 to 57.5 million which accounts for half of the U.S. population growth [21]. According to the Centers for Disease Control and Prevention [1], regular participation in physical activity is higher in C (23%) compared to H (16%). This disparity may lead to poorer health status in H adults due to the association between physical activity and health. In fact, incidence of metabolic syndrome is higher in Hispanic versus Caucasian women (36 vs. 24%) [22]. However, to our knowledge, no study has examined exercise responses in H adults, so it is unknown if they maintain a similar ability to improve VO_2_max in response to exercise training as C. Furthermore, women are less likely to be physically active as men, as 54% of men compared to 46% of women currently meet the Physical Activity Guidelines [1].

Clinicians and fitness professionals promote physical activity in their clientele to enhance fitness and health status, yet not everyone responds similarly to exercise training. For that reason, personalized exercise prescription [23] is becoming more popular to ensure that all persons reap the many benefits of exercise. However, the current Physical Activity Guidelines are ethnically blind, and a more recent iteration of this policy [24] states that research is needed to examine ethnicity as a potential mediator of responsiveness to regular physical activity. Data from a recent study by Alvarez et al. [25] showed significant increases in various cardiometabolic markers in Chilean adults of European and Latin American AmerIndian descent that were mostly similar between groups. To date, there are no studies investigating the effects of physical activity on cardiorespiratory fitness in Hispanics who comprise 18% of the U.S. population and may have increased health risks versus C [26]. Consequently, the purpose of this pilot study was to determine if ethnicity alters the change in cardiorespiratory fitness to a low dose of high intensity interval training in inactive women. We hypothesized that there will be a similar change in VO_2_max between Hispanic and Caucasian women, similar to findings from the HERITAGE study [11].

## Methods

### Participants

**Fig 1:**
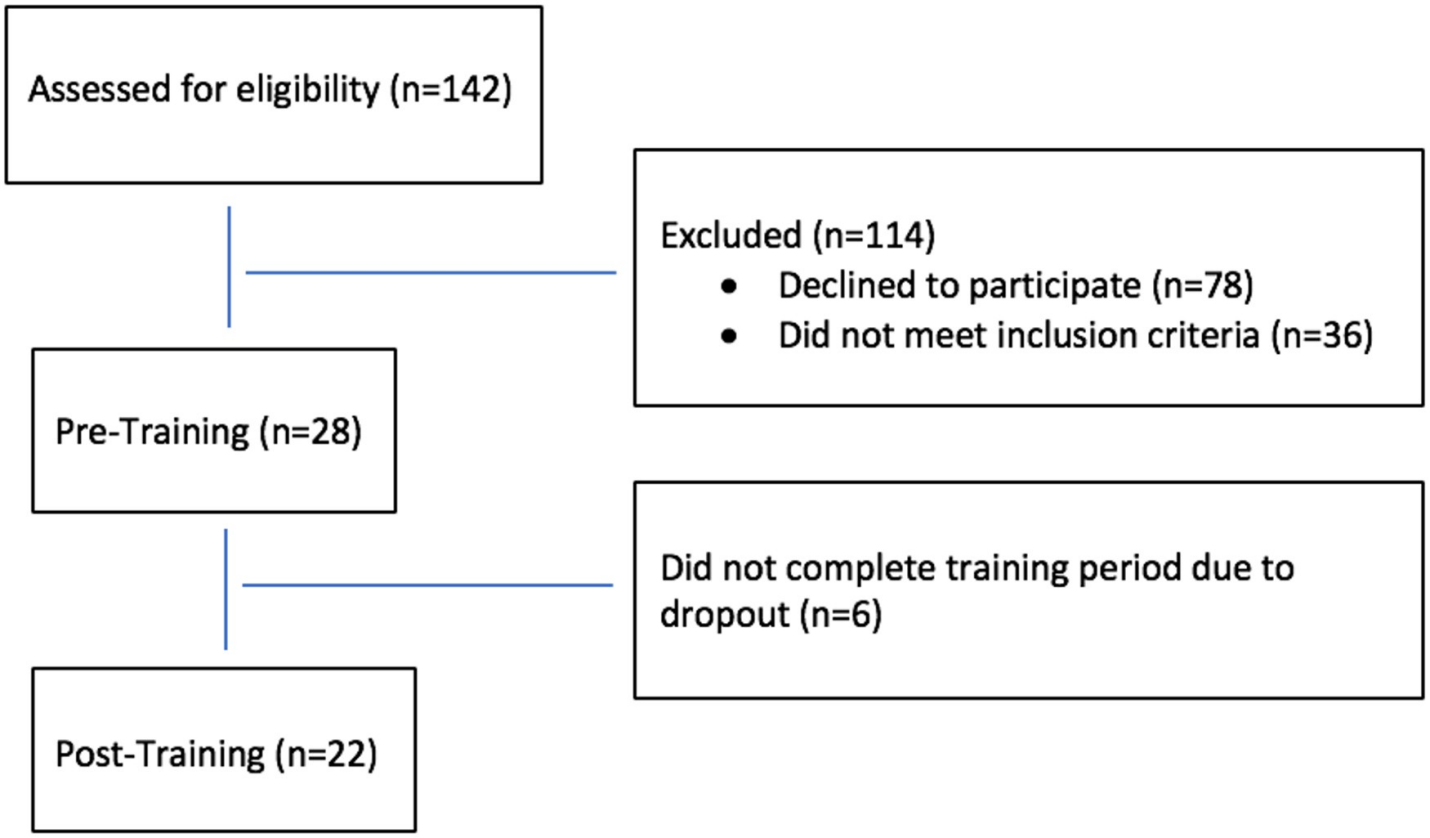
Participant flow through the study.

Twenty-eight healthy, inactive (< 150 min/wk of physical activity in the last year), non-obese (BMI < 30 kg/m^2^) women ranging from 19–35 years old initiated this study. Six women (2 C and 4 H) dropped out after initial testing due to lack of time to perform training, so 22 women completed the study. Participant recruitment, screening, and dropout are shown in Fig 1. Prospective participants were recruited through flyers posted at the University as well as through contacts of the Authors. The women were either 100% Caucasian (n = 12) or 100% Hispanic (n = 10), which was identified by self-report. To do this, we recorded the ethnicity of their parents and grandparents, and all had to have the same ethnicity for each woman to be included. Participants did not smoke, were not taking any medications, and had no physical condition that may modify response to training. Subjects completed a health history and physical activity questionnaire (IPAQ) to confirm that they met these guidelines. Participants provided written informed consent before participating in this study, whose protocol was approved by the CSU—San Marcos Institutional Review Board (S1 Protocol). Their physical characteristics are shown in Table 1. There was no difference (p > 0.05) in any baseline variable between subjects except for height. This protocol is now registered with clinicaltrials.gov (NCT 04449016), yet this registration was not done until after the trial was completed due to an oversight of the Primary Investigator.

**Table 1:** Participant physical characteristics (mean ± SD).

Parameter	Caucasian (n = 12)	Hispanic (n = 10)	*p*
Age (yr)	26.33 ± 5.65	22.60 ± 2.46	0.07
Height (cm)	164.79 ± 6.78	157.35 ± 5.61	0.01*
Mass (kg)	60.49 ± 7.40	60.56 ± 8.0	0.98
**Body mass index (kg/m^2^)**	22.2 ± 2.0	24.2 ± 2.3	0.20
VO_2_max (mL/kg/min)	31.07 ± 3.74	28.37 ± 3.83	0.12
Peak Power Output (W)	175.67 ± 27.14	164.20 ± 27.95	0.34
Physical Activity (steps/2 days)	20934.91 ± 3703.25	18562.67 ± 7491.68	0.37

* = p < 0.05 between Caucasian and Hispanic women

### Design

This study was a longitudinal, repeated measures between subjects design conducted from February 2018 to December 2019. Before each trial, participants were well rested and hydrated, not sick, and refrained from intense exercise for 24 hours and alcohol consumption for 48 hours. Trials were separated by a minimum of 24 hours, and the study was completed within a 5-week period. Baseline testing consisted of assessment of VO_2_max on days 1 and 2. Baseline tests were repeated to minimize learning effects and identify within-subject variability in our measures, as previously performed [27]. Participants were required to maintain their dietary habits during the study and not initiate any new regular physical activity outside the study. After training, VO_2_max was re-assessed following procedures described below, and held at least 24 h after the last training session.

### Assessment of VO_2_max

Using a brief survey, subjects were asked a series of questions to ensure that they met the pre-test guidelines for this assessment, which included being healthy, hydrated, well rested, had not performed strenuous lower body exercise in the previous 48 hours, and fasted for 3 hours prior. Initially, height and body mass were assessed using a balance scale and stadiometer. Each participant subsequently performed VO_2_max testing on an electronically-braked cycle ergometer (Velotron DynaFit Pro, Quarq, Spearfish, SD) using a ramp protocol. Individual adjustments on the cycle ergometer including seat height, seat slide, and handlebar height were adjusted for each participant during the initial trial and maintained for subsequent trials. The initial work rate began at 30 or 40 W for 2 minutes followed by a 15 or 20 W/min increase in power output until volitional exhaustion. During exercise, subjects expired through a plastic mouthpiece and low resistance valve into tubing connected to a mixing chamber. Measures of ventilation and expired fractions of oxygen and carbon dioxide were obtained throughout exercise by a metabolic cart (ParvoMedics True One, Sandy, UT). Gas exchange data including VO_2_, VCO_2_, and ventilation (V_E_) were time-averaged every 15 seconds. The test was terminated when the subject’s pedal cadence was below 50 rev/min. From this test, peak power output (PPO) was identified as the value coincident with exhaustion and was used to set intensities for HIIT, which was the average PPO from both trials. Ten minutes after the ramp test, the participant underwent a verification test to confirm VO_2_max attainment. After 2 minutes of cycling at 10% PPO, participants pedaled “all-out” at a work rate equal to 105% PPO. This constant load test performed at a work rate above that eliciting VO_2_max has been identified as a robust approach to confirm VO_2_max attainment in many populations [28, 29]. Baseline VO_2_max and related gas exchange data were identified as the average of all four values acquired, and post-training data were represented by the average of the ramp and verification value.

### Assessment of hemodynamic function

Initially, participants entered the laboratory and sat quietly for 5 minutes. Seated blood pressure was measured twice at the antecubital space using a blood pressure cuff and sphygmomanometer (Omron Health Care, Vernon Hills, OH). An alcohol swab was used to clean the neck, right chest, trunk at V6, and spine, and then an electrode gel (NuPrep, Weaver and Company, Aurora, CO) was rubbed into these areas and the skin further cleaned with a paper towel. Two sets of electrodes (Skintact ECG electrodes, Leonhard Lang GmbH, Innsbruck, Austria) were applied above the supraclavicular fossa at the left base of the neck and at the height of the xiphoid on the spine. Another pair of electrodes (one placed on the right chest and another at V6) were used to monitor the ECG trace. Once applied, these leads were taped to the skin to minimize movement. The participant sat on the cycle ergometer and remained silent and motionless, and the thoracic impedance device (Physioflow Enduro, Manatec, Strasbourg, France) was calibrated following a 30-beat procedure using the baseline BP value, which was averaged. Once resting values of HR, stroke volume (SV), and cardiac output (CO) were recorded, subjects were required to sit for an additional 60-seconds before the warm-up. SV, CO, and HR were recorded beat-by-beat and averaged every 15-seconds during exercise. Arterio-venous-O_2_difference (a-vO_2_diff) was calculated as a quotient of VO_2_ and CO and expressed in mL/dL. Maximal values were identified as the highest score at any time during exercise and was averaged across both baseline tests.

### Habitual physical activity

Participants were advised to maintain their inactive lifestyle during the study. They were given an accelerometer (Actigraph, Pensacola, FL) to wear on their wrist for a two-day period during baseline testing, a two-day period mid-way through the study, and a two-day period during post testing. Steps completed over the two days were used to quantify habitual physical activity.

### High intensity interval training

Each subject reported to the laboratory three times a week at the same time of day within subjects. Training was performed on the same electronically braked cycle ergometer. Prior to the session, women completed a 4-minute warmup at 10% PPO. Each subject completed nine sessions of low-volume HIIT over a 3-week period which consisted of eight to ten 1-minute bouts of cycling at 85% PPO with 75-second recovery between bouts at 10% PPO. This protocol is well-tolerated in inactive individuals [30]. A description of the protocol is detailed in Table 2. Continuously during each session, heart rate was monitored using telemetry (Polar, Lake Success, NY). On days 1, 3, 6, and 9 of training, the physical activity enjoyment scale (PACES) was completed [31].

**Table 2:** Description of the low-volume high intensity interval training regimen used in the present study.

Week	Number of Bouts	Bout Duration (seconds)	Recovery Duration (seconds)	Intensity (% PPO)	Total Time (minutes)
1	8	60	75	85	22
2	9	60	75	85	24.25
3	10	60	75	85	26.5

### Assessment of typical error

Typical error (TE) was computed using data from two days of baseline testing with the following equation: TE = SD_diff_/√2. In this equation, SD_diff_ is the standard deviation of the change scores between the two repeated tests. Non-response for each outcome was defined as an individual who failed to demonstrate a change greater than TE (0.11 L/min and 1.4 L/min for VO_2_max and CO, respectively). This approach was used as it represents a threshold past which the odds of a real change are 12:1 [32].

### Statistical analysis

Data are expressed as mean ± SD and were analyzed using SPSS Version 24.0 (Chicago, IL). The Shapiro–Wilk test was used to assess normality and the Mauchly’s test was used to assess sphericity, and these assumptions were met for all dependent variables including the main outcomes equal to VO_2_max, PPO, and CO. Independent t-test was performed to identify differences in demographic and physiological variables between groups at baseline. Repeated two-way ANOVA (time = pre versus post, group = C versus H) with repeated measures was performed to identify differences in VO_2_max, hemodynamic, and gas exchange variables. The Greenhouse–Geisser correction was used to account for the sphericity assumption of unequal variances across groups. If a significant F ratio occurred, Tukey’s post hoc test was used to identify differences between means. Cohen’s d was used as an estimate of effect size [33]. Statistical significance was set as p < 0.05. Our sample size per group is similar to previous research comparing efficacy of HIIT in young adults completing different exercise regimens [5, 30, 34]. In addition, data show that an increase in VO_2_max as small as 1.5 ml/kg/min is clinically meaningful [35].

## Results

### Participant flow

**Fig 2:**
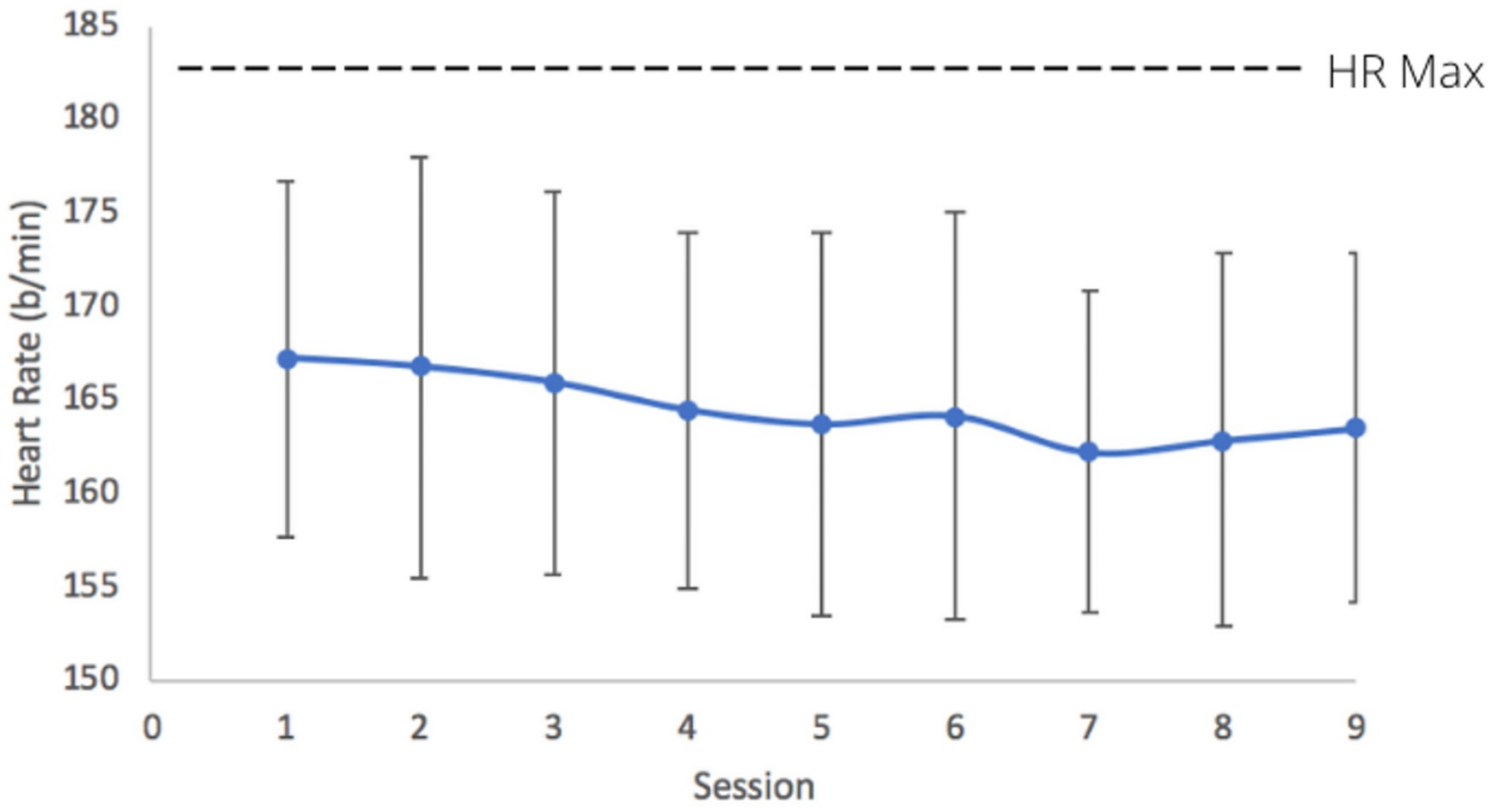
Heart rate response to nine sessions of low-volume high intensity interval training in Hispanic and Caucasian women (mean ± SD).

Our final sample size is somewhat lower than that initially proposed which included 15 individuals of each ethnicity. Unfortunately, we faced issues with participant retention and dropout and in addition, a relatively low number of people were interested in serving as a subject in this study. Initially, we had intended to recruit men and women to participate in this study to more efficiently meet our intended sample size, yet few men expressed interest in participating, so ultimately, we restricted participation to solely women. There was 99.5% compliance to HIIT as women performed 197 of 198 sessions of training. The average peak heart rate during training was equal to 164.6 ± 9.9 b/min (89.8% of HRmax), which verifies the intensity of HIIT (Fig 2). Enjoyment as measured with PACES did not change from session 1 to 9 (88.2 ± 17.2 versus 90.5 ± 19.5, p = 0.37) and there was no effect of group (p = 0.40) or group by training interaction (p = 0.34). Results showed no change in habitual activity (p = 0.19) from pre- to post-training as well as no group by training interaction (p = 0.86) or effect of group (p = 0.74).

### Change in VO_2_max and gas exchange data

**Fig 3:**
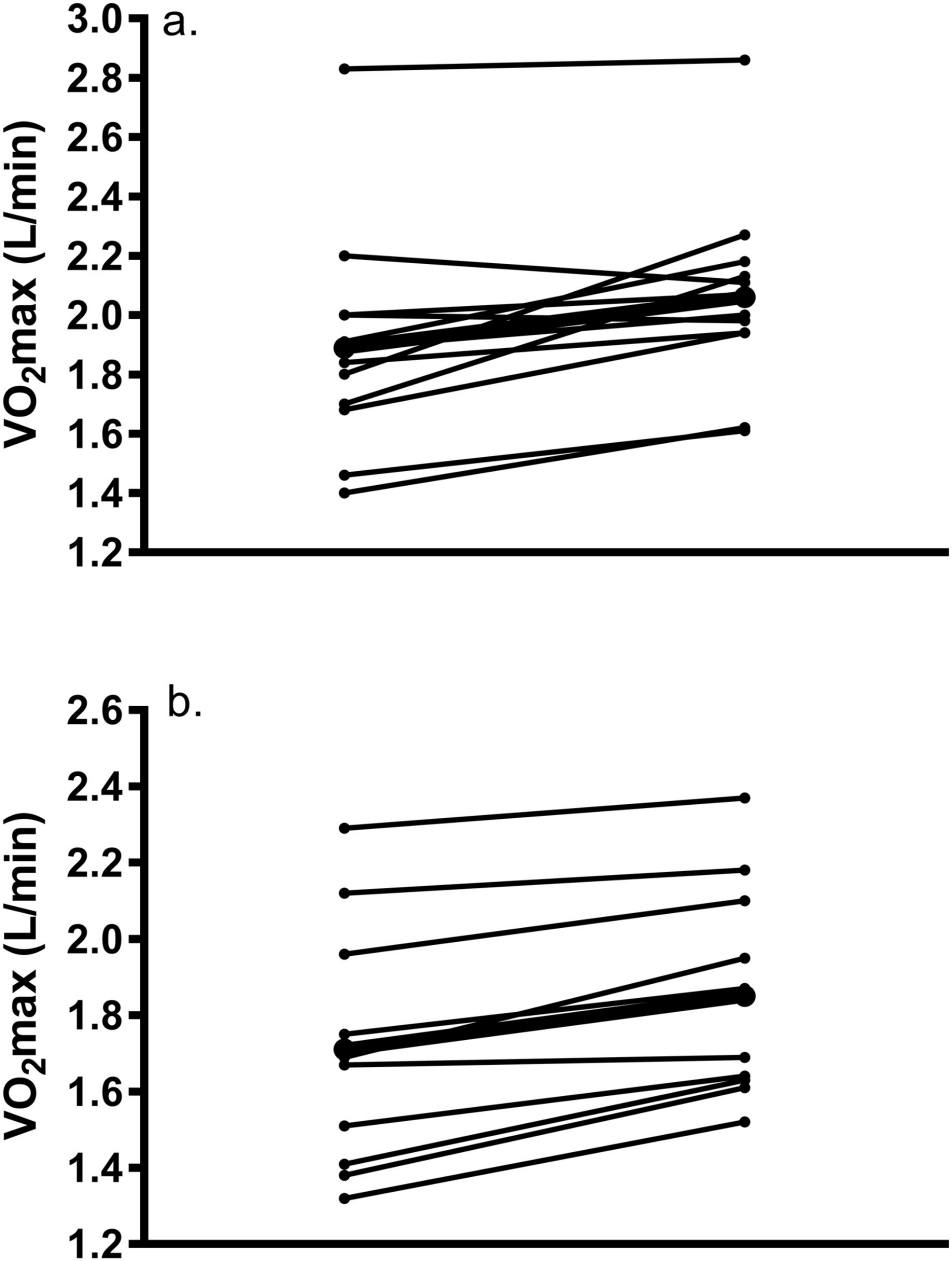
Individual response of change in absolute VO_2_max from pre- to post-training in a) Caucasian and b) Hispanic women; dark line represents the mean response.

Changes in gas exchange data, PPO, and HRmax are revealed in Table 3. Results showed a significant main effect of training for both relative (F_1,20_ = 13.97, p = 0.001) and absolute VO_2_max (F_1,20_ = 27.29, p< 0.001), but there was no group by training interaction (p = 0.65 and p = 0.66) or effect of group (p = 0.06 and 0.17). Relative and absolute VO_2_max was increased by 8.4 ± 12.3% and 9.9 ± 9.9% in C and 7.8 ± 7.3% and 7.6 ± 6.2% in H. Results showed a large effect for the change in relative VO_2_max in C (d = 1.32) and H (d = 1.04) as well as absolute VO_2_max (d = 1.78 and d = 1.48). Post hoc analyses showed that the post-training value was higher than at baseline for both C and H. Significant increases were also exhibited in PPO (p<0.001, d = 2.82 and 2.61), VCO_2_ (p<0.001, d = 1.72 and d = 1.33), and V_E_ (p = 0.008, d = 0.99 and d = 0.88). There was no effect of group or group by training interaction for PPO (p = 0.28 and 0.72), VCO_2_ (p = 0.11 and 0.55), or V_E_ (p = 0.51 and 0.84). Individual changes in absolute VO_2_max are demonstrated in Fig 3. Our data revealed that 64% of participants (eight C women and 6 H women) showed a meaningful change (greater than TE of 0.11 L/min) in absolute VO_2_max from baseline.

**Table 3:** Change in gas exchange and hemodynamic variables in response to nine sessions of low-volume HIIT (mean ± SD).

Parameter	Caucasian (n = 12)			Hispanic (n = 10)		
	Pre	Post	ES *(d)*	Pre	Post	ES *(d)*
VO_2_max (mL/kg/min)	31.07 ± 3.74	33.70 ± 3.74*	1.32	28.37 ± 3.83	30.45 ± 3.64*	1.04
VO_2_max (L/min)	1.89 ± 0.37	2.06 ± 0.32*	1.78	1.71 ± 0.33	1.85 ± 0.29*	1.48
VCO_2_max (L/min)	2.23 ± 0.38	2.49 ± 0.38*	1.72	2.02 ± 0.36	2.22 ± 0.30*	1.33
V_E_max (L/min)	80.66 ± 14.37	89.38 ± 15.36*	0.99	75.83 ± 24.30	83.45 ± 24.93	0.88
HRmax (b/min)	182.25 ± 9.72	184.04 ± 10.59	0.54	184.75 ± 6.0	184.55 ± 5.88	0.06
Peak Power Output (W)	175.67 ± 27.14	194.21 ± 21.81*	2.82	164.20 ± 27.95	181.20 ± 28.55*	2.61
Stroke Volume (mL)	97.77 ± 11.09	109.23 ± 13.83*	1.63	99.73 ± 11.22	100.17 ± 7.44	0.06
Cardiac Output (L/min)	17.82 ± 2.31	19.92 ± 2.47*	1.66	18.41 ± 2.42	18.52 ± 1.35	0.09
a-vO_2_difference (mL/dL)	10.59 ± 1.22	10.40 ± 1.40	0.28	9.32 ± 1.53	10.01 ± 1.43	1.02

Asterisk (*) denotes a significant difference (p < 0.05) compared to pre-intervention measure within group. ES = Effect Size.

### Change in hemodynamic data

**Fig 4:**
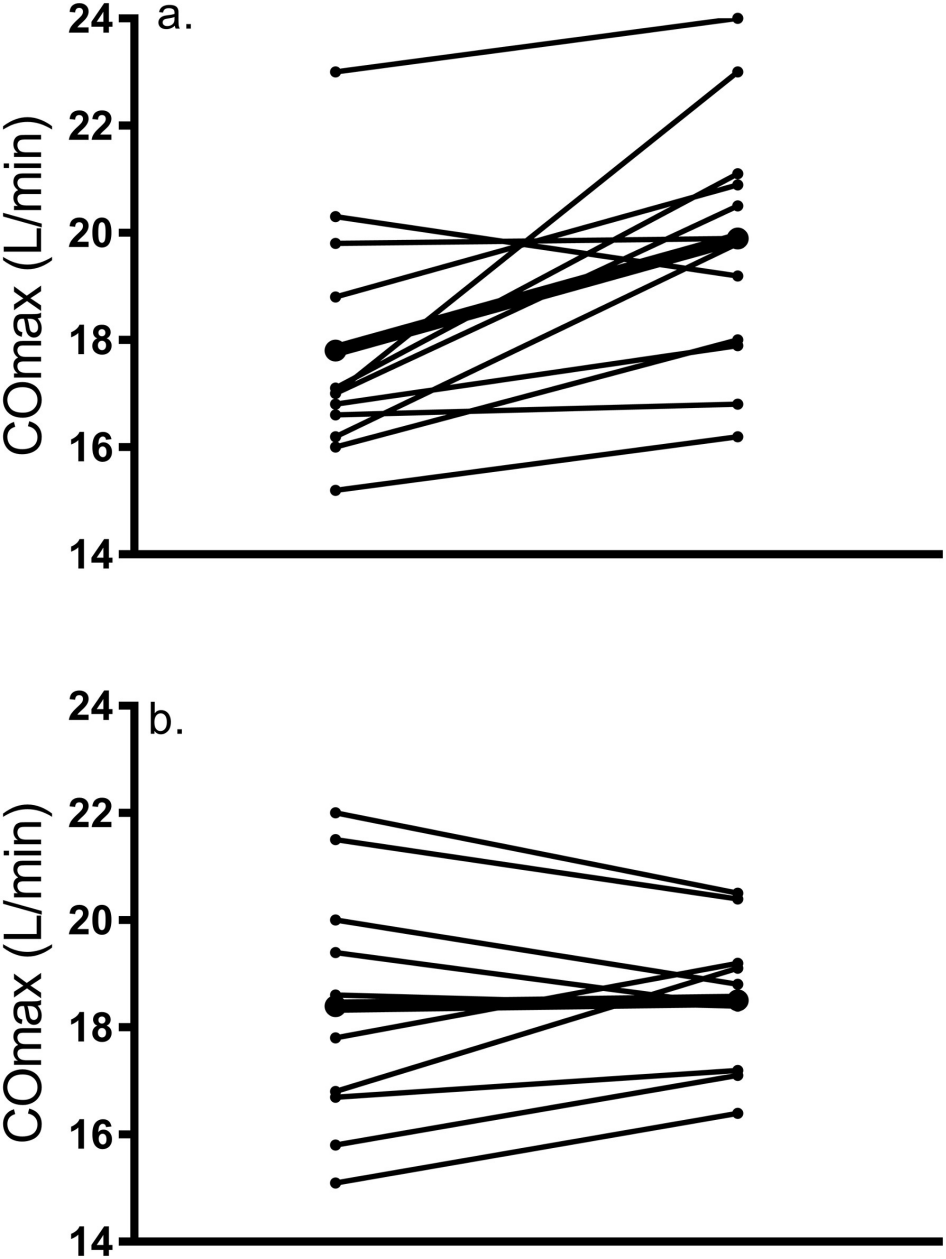
Individual response of change in maximal cardiac output from pre- to post-training in a) Caucasian and b) Hispanic women; dark line represents the mean response.

**Fig 5:**
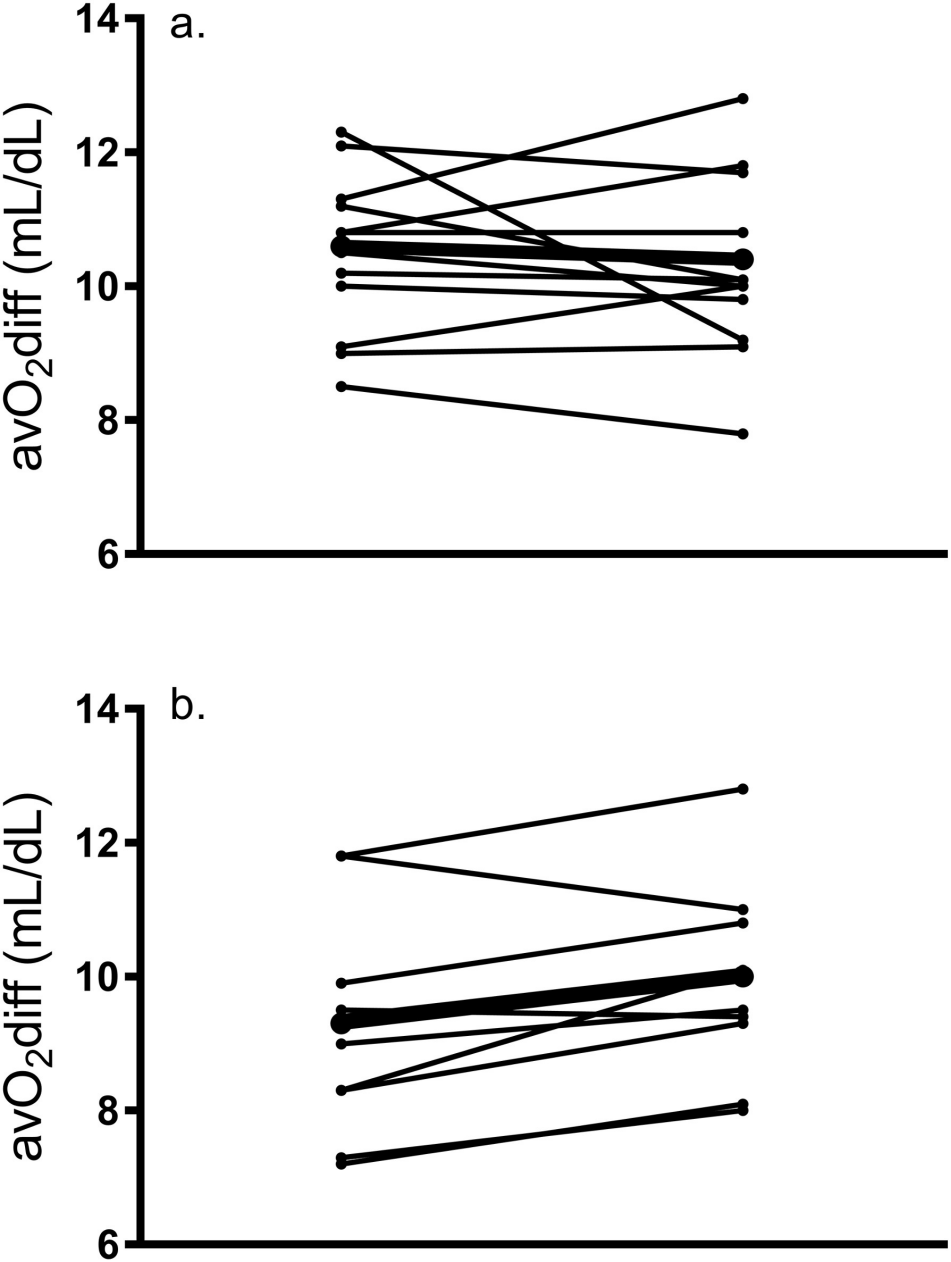
Individual response of change in a-vO_2_diff from pre- to post-training in a) Caucasian and b) Hispanic women; dark line represents the mean response.

These data are revealed in Table 3. Maximal HR did not change in response to training (p = 0.46) and there was no main effect of group (p = 0.68) or group by training interaction (p = 0.36). There was no effect of group (p = 0.64), but results showed a significant main effect of training for CO (F_1,20_ = 7.57, p = 0.012) and a group by training interaction (p = 0.02) as CO was increased by 11.78% in C, which was higher than the change seen in H (0.59%). Post hoc analyses showed that the post-training CO was significantly higher than at baseline in C (d = 1.7) but not H (d = 0.1). Our data revealed that 32% of participants (six C women and one H woman) showed a meaningful change (greater than TE of 1.4 L/min) in cardiac output from baseline. A similar main effect of training (F_1,20_ = 7.16, p = 0.015) and group by training interaction (p = 0.022) were revealed for SV, but no effect of group (p = 0.46). Post hoc analyses showed that post-training SV was significantly higher than baseline in C (d = 1.7) but not H (d = 0.2). There was no main effect of training on a-VO_2_diff (F_1,20_ = 1.32, p = 0.26) or effect of group (p = 0.14) but there was a tendency for a group by training interaction (p = 0.054), and a large effect size was seen in H (d = 1.02) but not C (d = 0.3). Individual changes in CO and a-VO_2_diff are demonstrated in Figs 4 and 5, respectively.

## Discussion

This pilot study compared changes in VO_2_max and cardiac output in inactive Caucasian and Hispanic women in response to nine sessions of low volume high intensity interval training. Our aggregate results show that both groups experienced comparable increases in VO_2_max, supporting data acquired in inactive [4] and active adults [36] performing HIIT. However, C women increased VO_2_max via an increase in CO; whereas H women achieved this via an increase in a-VO_2_diff. These results, albeit preliminary, suggest discrepant hemodynamic responses to low-volume HIIT in women of different ethnicities.

Results from a systematic review [6] report a mean improvement in VO_2_max equal to 6% in response to 2–10 weeks of HIIT. Our increase is slightly larger (8% in C and H) which is attributed to the lower baseline fitness of our sample. Despite the overall increase in VO_2_max, there was large variability in this outcome (Fig 3), and 4 of 22 participants showed either no increase or a decline in VO_2_max in response to training. This finding supports previous research revealing that not all participants display meaningful increases in VO_2_max in response to HIIT of lower intensities when compared to higher intensities [27, 36, 37]. Conversely, Astorino et al. [30] and Matsuo et al. [34] concluded that the change in VO_2_max was similar in response to discrepant intensities of low-volume HIIT, yet these were longer duration studies (8 to 12 weeks compared to 3 weeks), and it is plausible that the greater training volume led to these similar increases. Due to these conflicting data, it is important to explore other variables mediating the response to training, as Bouchard et al. [11] determined that 50% of response to training is likely due to factors other than genetics. Alternative explanations for the individual responsiveness to training include discrepancies in autonomic function [38], quality of physical and nutritional recovery after each session [15], and the institution of training according to %PPO rather than metabolic thresholds, which places participants at different levels of homeostatic stress [15].

Despite many studies exhibiting increases in VO_2_max in response to HIIT, there is a paucity of data concerning the specific adaptations leading to this increase in cardiorespiratory fitness. Central (CO) and peripheral (a-VO_2_diff) adaptations to training are responsible for the improvement in cardiorespiratory fitness [4], yet previous research is equivocal regarding the specific contributions leading to these improvements. Our results seen in C are supported by Astorino et al. [36] who reported that significant increases in CO after 3 weeks of low-volume HIIT in active men and women are due to increased stroke volume rather than alterations in a-VO_2_diff. Similarly, Warburton et al. [39] showed that increased left ventricular function (SV) and increases in vascular volumes are responsible for the increase in VO_2_max after 12 weeks of interval training. Despite the change in a-VO_2_diff being non-significant, there was a large effect seen in H, suggesting that this variable was likely responsible for the significant increase in VO_2_max. Our findings are supported by Daussin et al. [4] who showed increases in a-VO_2_diff in inactive adults performing 8 weeks of continuous and interval training, yet significant increases in CO were only elicited after interval training.

Previous research [19] identified distinct differences in various lipoprotein subclasses between C and AA adults, which supports the need to determine if ethnicity contributes to some of the individual response to training. Prior results [40] acquired in Caucasian and African American men and women exhibited no difference in VO_2_max between groups in response to 20 weeks of MICT. Yet, little is known about changes in cardiorespiratory fitness and hemodynamic responses in Hispanics who comprise an ever-expanding portion of our population. In a comparative study examining the cardiac response to exercise between Indigenous and European adults, Foulds et al. [41] revealed that both groups show similar responses to maximal exercise, yet only Indigenous adults exhibited a significant cardiac response to submaximal exercise. Their findings suggested that Indigenous adults are not able to recover as quickly as European adults, which may increase risk of cardiac dysfunction. Recently, Jones et al. [42] examined various responses to exercise between Europeans, South Asian, and African Caribbean individuals with type-2 diabetes. Results showed that the South Asian and African Caribbean adults had reduced exercise capacity compared to the Europeans, suggesting that ethnicity may mediate some of the differences in body composition, glycemic responses, and cardiorespiratory fitness. Our data show no effect of ethnicity on the VO_2_max response to HIIT, yet C and H may experience increases in VO_2_max due to different mechanisms, as C exhibited adaptations representing improved O_2_ delivery; whereas, H revealed increases in O_2_ extraction.

To our knowledge, this is the first HIIT study examining the effect of ethnicity on these outcomes, however, due to our small sample size, we may have been underpowered to detect any significant differences between groups. Also, this particular regimen may be inadequate to elicit a marked difference in adaptation between groups due to the limited duration and volume of training. Our data do not apply to women with chronic disease or those who are obese. We did not measure body composition at baseline, so we do not know if women differed in percent body fat or muscle mass which may alter training responsiveness. Moreover, no measure of autonomic function was acquired which is related to the VO_2_max response to training [38]. Lastly, ethnicity was identified through self-report as used in a prior study [41], yet a DNA test may be a more appropriate test to confirm this trait in our women. However, the study is strengthened by repeated assessment of baseline cardiorespiratory fitness and inclusion of verification testing of VO_2_max, which reduces error in our pre-training measures and follows best practice for training studies [43]. During the study, we monitored variations in habitual activity which is related to the change in VO_2_max with training [44], and our data show no change in habitual physical activity. Lastly, all training was supervised and resultant intensities met the definition of HIIT.

## Conclusion

Our results demonstrate the viability of low-volume HIIT in Hispanic women and show that a small dose of training led to significant increases in VO_2_max similar in magnitude to Caucasians. Future research should include a more in-depth analysis of subjects’ genetics and increase the volume or duration of training to better elucidate potential effects of ethnicity on training adaptation.

## Supporting information

S1 Checklist TREND statement checklist. (PDF)

S1 Data (XLSX)

S1 File (DOCX)

S1 Protocol (PDF)
