# Supplementary material for: Changes in VO2max and cardiac output in response to short-term high-intensity interval training in Caucasian and Hispanic young women: A pilot study
Source: PLoS One. 2021 Jan 22;16(1):e0244850. doi: 10.1371/journal.pone.0244850 (PMC7822506; doi:10.1371/journal.pone.0244850)
Supplement: S1 File — (DOCX) [file pone.0244850.s003.docx]

***
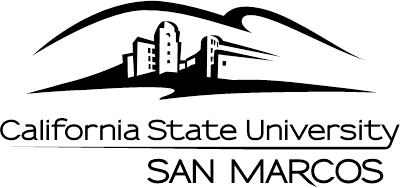
College of Education, Health and Human Services***

***Todd A. Astorino, Ph.D.***

***Professor, Kinesiology***

*California State University San Marcos, San Marcos, California 92096-0001 USA*

***Email:*** [*astorino@csusm.edu*](mailto:astorino@csusm.edu) ***Tel:*** *760-750-7351* ***Fax:*** *760-750-3190*

**Informed Consent—Effect of ethnicity on adaptation to short-term high intensity interval training (HIIT)**

Dr. Todd A. Astorino, Professor, Department of Kinesiology, is conducting a study to examine if your ethnicity may alter potential changes in health-related outcomes in response to HIIT. This study is merited as data show that some people are “high” or “low” responders to exercise training, and ethnicity has been identified as a potential cause of this varying response. You were recruited for this study because:

1. you are physically inactive (< 1 hour/week of physical activity in the last 12 months) and live in the greater San Diego County region

2. you are healthy, non-obese, and free from any injury and do not take any medication which may affect your responsiveness to exercise training

3. you are a male or female ages 18 – 45 years old

If you meet these criteria and choose to volunteer for this **5-week** study, you will do the following:

**METHODS**:

On the first day, you will arrive at Academic Hall 115 on the campus of CSU—San Marcos after not eating for 2 hours and being well rested and hydrated. You will also need to wear shorts/sweatpants and a sports bra if you are a woman, and be shaven and shirtless if you are a man. You will perform one bout of stationary cycling to determine your aerobic fitness (VO2max). The intensity of cycling will be steadily increased during this 8 – 12 minute bout and you will be asked to exercise to fatigue. After a brief rest, you will complete an additional bout of intense exercise to confirm that you achieved VO2max. During this bout, you will breathe into a valve and wear headgear and noseclips to allow your calorie expenditure to be measured. A plastic strap will be placed around your trunk to measure heart rate. Also during this session, a portable device will be used to determine changes in heart function, which will require you to have electrodes placed on your neck (2), spine (2), and trunk (2, right chest and left side below your heart). This will require women to wear a sports bra and men to shave their neck and chest and perform this bout shirtless. **This session will take approximately 50 minutes.** You will repeat this test at least 3 days later at the same time of day to provide a stable baseline value of your fitness (VO2max), which will take approximately 50 minutes**.**

On 2 subsequent sessions, you will come to the lab after an overnight (> 10 hours) fast for measurement of how much fat and carbohydrate you burn. This measure has been shown to be linked to weight gain (Colberg et al. 1995) as well as health risk in adults (Robinson et al. 2015). After a 6 minute warmup at a low intensity, you will complete four 4 minute stages at 20, 30, 40, and 50 % of your maximal intensity on the stationary bike, during which you breathe into a three way valve and wear the headgear. Blood will be drawn from a fingertip (20 microliters, about the size of a pinhead) pre- and post-exercise as well as at one point during this bout using a lancet and portable monitor. Standardized procedures for blood collection will be followed.

**This bout will take approximately 40 minutes.**

You will return at the same time of day at least 24 hours later and complete day 1 of HIIT, consisting of 8 1 min bouts of intense exercise on the stationary bike interspersed with 1 min of recovery at a light intensity. **All training sessions will take < 30 minutes and will be held 3 days per week for 3 weeks at the same time of day.** On days 3, 6, and 9 of training, Rating of Perceived Exertion (RPE, Borg CR10), affect (+5 to -5, Hardy & Rejeski 1991), and Physical Activity Enjoyment (PACES) will be assessed using brief surveys. Training sessions 2-3 will mirror the exercise regime of day 1; whereas, days 4-6 will require 9 bouts at the same intensity, and days 7-9, you will perform 10 bouts. During all sessions, heart rate (HR) will be assessed continuously using the HR monitor. **You will be required to abstain from any current physical activity and maintain your dietary intake during the study**, which will be verified with a written log.

Approximately 48 hours after the 9^th^ and final training session, you will repeat the baseline assessment of your aerobic fitness (VO2max), following the required pre-test guidelines, and then at least 48 hours later, repeat the test of fat and CHO burning.

**RISKS**: Potential risks of participation in this study include:

1. There is potential for soreness and/or fatigue resulting from the exercise bouts, as well as potential for nausea and/or dizziness as well as small onset of injury.

2. During VO2max testing, there is a small risk of cardiac event. In addition, you may feel uncomfortable during the measurement of cardiac function.

3. Potential for coercion to participate in the study.

4. Potential loss of time, financial gain, or employment due to the time demands of the study.

5. Potential for your identity to be revealed through participation in the study.

6. Potential for breach of confidentiality of data.

7. Chance of slight pain or bruising to be experienced during the finger stick blood samples.

8. Potential for embarrassment during the fitness test as you will wear a sports bra if you are a woman and be shirtless if you are a man.

**SAFEGUARDS**: These are in place to minimize the potential for risks experienced by you, and include:

1. Soreness, leg pain, nausea, and/or fatigue will be minimized by requiring subjects to complete a cool-down after each bout of exercise, as previously used (Burgomaster et al., 2006; 2007). Moreover, you are healthy so any incidence of this will be brief and likely of minimal magnitude. Similar procedures to these were used in many recent studies in persons performing HIIT in my Lab without incident (Astorino et al. 2017a, 2017b; Thum et al, 2017; Wood et al. 2016, etc.), so chance of injury is unexpected.

2. Potential for cardiac events during the VO2max test will be minimized by using the health-history questionnaire to screen you for possible increased risk, which would exclude you from participating in the study. Risk of cardiac events in older inactive persons with elevated health risk is approximately 1 in 20,000, so the risk in young active people is miniscule. And there is no alternative to assessment of cardiac function, although it will be performed with only members of the Research team present.

3. You will not be coerced in any way to partake in the study, and you will be reminded that you can withdraw from the study at any time without consequences, and only the Primary Investigator will be aware of your choice to stop participation.

4. Participation is approximately **2 hours per week over 5 weeks**, so this is not excess demand on your time. Moreover, researchers will do their best to make all visits to the Lab as *smooth* and *time-efficient* as possible.

5-6. Participants' names on all documents (data sheets, etc.) will be replaced by a code developed by the Primary Investigator to conceal your identity. Data-containing folders will be placed in locked cabinets only accessible to the PI.

7. Any pain experienced during the fingerstick procedure is brief, and the volume of blood drawn is extremely minute to have no impact on your health status.

8. The lab door will be closed and only members of the Research team will be present during this bout, which will somewhat reduce potential for embarrassment.

**BENEFITS**: Study benefits include assessment of your aerobic fitness and fat and CHO use which can be used to gauge your health status, and it is likely that completion of these bouts will give you greater self-confidence in performing intense exercise, and likely gains in fitness too. If our data show a sizable difference in adaptation based on ethnicity, it will demonstrate the importance of personalizing exercise regimes to improve overall health status. Also, these data will add to the growing body of knowledge concerning how to optimize efficacy of exercise regimes employing HIIT.

**FINANCIAL EXPENDITURES AND INCENTIVES**: There is no cost for you to participate in this study. We will also pay you $125.00 upon completion of all requirements of the study, to compensate you for your time and effort.

**VOLUNTARY PARTICIPATION**: Participation in this study is voluntary, and you may withdraw from the study at any time without penalty of any kind. No one other than the Primary Investigator will know of your choice to stop participating in the study.

**CONTACT INFORMATION**: The Primary Investigator will gladly answer any questions that you have regarding this study. If you have further questions, please contact Dr. Todd A. Astorino by phone at (760) 750-7351 or via email at astorino@csusm.edu.

This study has been approved by the California State University, San Marcos Institutional Review Board. Questions about your rights as a research participant should be directed to the Chair of the Institutional Review Board at (760) 750-4029. You will be given a copy of this form to keep for your records.

☐ I agree to participate in this research study.

_________________________________________ ____________________

Participant’s Name Date

________________________________________

Participant’s Signature

________________________________________ ____________________

Researcher’s Signature Date
