# Supplementary material for: Changes in VO2max and cardiac output in response to short-term high-intensity interval training in Caucasian and Hispanic young women: A pilot study
Source: PLoS One. 2021 Jan 22;16(1):e0244850. doi: 10.1371/journal.pone.0244850 (PMC7822506; doi:10.1371/journal.pone.0244850)
Supplement: S1 Protocol — (PDF) [file pone.0244850.s004.pdf]

EXPEDITED REVIEW—2-16-18

Effect of ethnicity on adaptation to short term high intensity interval training (HIIT)

PI: Todd A. Astorino Ph.D KINE-CEHHS

Phone: -7351 email: astorino@csusm.edu

1. Purpose of project and background: Regular participation in moderate intensity continuous training (MICT) improves fitness and health status in adults (Garber et al. 2011), yet participation in physical activity is low (~ 20 % of adults, CDC 2016). One barrier to regular physical activity is lack of time (Trost et al. 2002), as the current Physical Activity guidelines recommend 30 minutes/day of MICT on most days of the week to reap the benefits of physical activity. Recently, an alternative to MICT, high intensity interval training (HIIT), has been shown to elicit similar (Nybo et al. 2012) and in some cases superior adaptations versus MICT (Milanovic et al. 2015) while being more time efficient. One defining trait of HIIT is that its intensity is higher than MICT in that repeated bursts of exercise (20 seconds to 4 minutes) at near-maximal to supramaximal intensities are completed and separated by recovery. In some cases, HIIT is viewed as more pleasant than MICT (Astorino and Thum 2017; Thum et al. 2017) which may make it feasible for many individuals and promote long-term adherence to this form of exercise (Williams et al. 2011).

Despite the documented efficacy of HIIT, we (Astorino & Schubert 2016) and others have shown individual responses to HIIT in that some people reveal marked adaptation; whereas, others reveal little to no adaptation to the identical HIIT regime. This is concerning especially when physical activity is used to combat severity or onset of chronic disease. Approximately 50 % of this heterogeneity is explained by genetics, yet the other 50 % is unknown and likely related to participants' sleep habits, dietary practices, habitual physical activity, as well as traits of exercise training (Mann et al. 2015). Recently, Physiologists (Buford et al. 2014) have emphasized that exercise training needs to be personalized to individuals so that EVERYONE can reap the health and fitness-related benefits of regular physical activity. However, scientists still do not understand why this individual response to exercise training occurs, so further research is needed.

In the late 1990s, Physiologists at multiple Universities initiated the HERITAGE study, whose goal was to examine genomic responses to 20 wk of standardized MICT in Caucasian (C) and African American (AA) adults (N ~ 700). Data showed that most adults respond to exercise training, although as stated above, individual responses were apparent and partially linked to ethnicity. Subsequent large-scale training studies (Slentz et al. 2008., etc.) examining the effect of exercise on various health-related markers rarely included populations other than C or AA, which suggests that much remains to be known whether other ethnicities respond similarly to chronic physical activity. Recently, Gill et al. (2014) stated that the minimum level of physical activity needed to confer health benefits across persons of various ethnicities may not be equal.

Moreover, they state that innate differences in cardiorespiratory fitness and capacity for fat oxidation potentially contribute to ethnic differences in the cardiometabolic risk profile, and that ethnicity-specific physical activity guidelines are warranted. It is evident that Physical Activity levels are typically lower in Hispanics compared to Caucasians (CDC 2017), which may impact their overall health status and may make them more susceptible to chronic disease. Overall, further work is needed to examine if equal doses of physical activity result in similar responses in persons of various ethnicity, which at this point is poorly understood.

This pilot study will answer the question if ethnicity alters physiological responses to a small dose of exercise training. We will implement 9 sessions of HIIT over a 3-week period in 2 groups: Caucasians (C), and Hispanics (H) to examine if ethnicity does alter responsiveness to exercise training.

2. Recruitment Procedures & Participant Population

A) List the expected number of participants for each population group included in this study. 30 total, 15 C and 15 H.

B) Describe all characteristics relevant to being selection of participants. (e.g., demographics, ethnicity, vulnerabilities, etc.)

Explain why you are targeting this specific population. Individuals will be healthy, sedentary (< 1 h/week of physical activity in the last 12 mo), men and women matched for age, body mass index (mass/height), and aerobic fitness across C and H. They will be ages 18-45 yr, non-obese, and free of disease or any ailment which may alter their ability to perform training as required in this study.

C) Indicate whether anyone might be excluded from participating and explain why. Prospective participants excluded from participation will include:
People who do not meet the inclusion criteria outlined above will be excluded i.e. persons who are older than 45 yr, unhealthy, obese, active, or do not fall into these 2 ethnicities. Persons of mixed race will also be excluded.

D) How will you find, recruit, or identify potential subjects? How will you select, from the volunteers, the final group of participants? Submit flyers, posters, or other oral or written invitations used to recruit potential participants.
They will be recruited through contacts of the Primary Investigator, via the CSUSM News Center post, and word of mouth. Anyone who expresses interest in participating must meet all inclusion criteria listed above to be eligible.

E) Will you be offering an incentive? Yes.
If yes, please explain procedure for any incentives that will be offered. Include how much participants must do to be eligible to receive credit. --To encourage participation, participants will be offered a stipend of $125.00 to complete all requirements of the study, which totals approximately 10 – 12 hours of their time. They will not be paid this amount unless they finish

the study, as their data cannot be used if they withdraw at any time due to limitations of our statistical packages.

3. Informed Consent Process.
Explain for each population participating in your research.
See the IRB web page on Informed Consent. See also Language Requirements.
A) How and when will you explain the study and the required elements of Informed Consent? Will you be doing this or will it be handled by a research assistant?
The PI will initially explain all aspects of the informed consent process over email and/or phone with all potential participants. At this time, they will also receive a health-history survey to allow the PI to gauge their health/fitness status and ensure they meet all inclusion-exclusion criteria. The informed consent document will be provided by the PI via email before the individual completes the first session in the lab; this is done to not waste any participant's time. The procedures outlined in the consent form will be discussed in detail on each day of data collection to ensure adequate knowledge and understanding of all inherent procedures followed in the study.

B) How much time will participants have to consider participating between the explanation described above, the receipt of the consent document, and the beginning of study?
A minimum of 1 week.

C) If there are subjects under the age of 18, how will the study be explained to them? How will parental consent and child assent be handled?
No subjects will be included in this study if they are <18 years of age.

D) If you are requesting a Waiver of Consent or a Waiver of Documentation of Consent, explain why this waiver is needed. Outline alternative procedures for obtaining consent or providing study information (e.g., information sheet, introduction screen for web survey, etc.).
N/A

E) Indicate the primary language(s) of your participants. If any participants is not fluent and comfortable with English, explain how you will ensure that participants' understanding of the activity for which they are giving consent.
The primary language of the participants is English. If a participant is not fluent and comfortable reading and understanding written and spoken English and they lack a qualified interpreter, they will not be included in this study.

4. Procedures and Methodology: Provide descriptions of each distinct procedure and each population group.
A) Provide a step-by-step explanation of your research activities and methodologies that involve human subjects. Be thorough.
We will implement 9 sessions of HIIT over a 3-week period in 2 groups: Caucasians (C) and Hispanics (H), with these groups comprising a large portion of the North County San Diego population. Ethnicity will be self-reported and we will exclude individuals who are mixed-race. Fifteen healthy, sedentary adults ages 18 – 45 (equal # of men and women who have completed < 150 minutes/week of exercise in the last year) will comprise each group. On day 1, height and

weight will be recorded, and VO2max will be determined using ramp cycling to exhaustion and simultaneous gas exchange data, followed by a verification test at 105 %PPO, which is used to verify VO2max attainment. Testing will require them to breathe into a three-way valve and wear headgear and noseclips. Participants will be rested, hydrated, post absorptive by at least 2 h, and dressed in exercise attire including shorts/tights and a t-shirt for this session, with their status confirmed with a brief questionnaire completed upon arrival to the Lab. Also during this session, Physioflow will be used to determine changes in stroke volume and cardiac output, which will require each participant to have electrodes placed on his-her neck (2), spine (2), and trunk (2, right chest and left side below nipple). This will require women to wear a sports bra and men to shave their neck and chest and perform this bout shirtless. Variables obtained from this test will include VO2max, HRmax, peak power output (PPO), and ventilatory threshold (VT, Caiozzo et al. 1986). This session will take approximately 50 minutes. They will repeat this test at least 3 d later at the same time of day to provide a stable baseline value of VO2max and allow us to determine individual variability in this measure.

On 2 subsequent sessions, they will come to the lab after an overnight fast for measurement of fat-carbohydrate oxidation (FatOx and CHOOx). This measure has been shown to be linked to weight gain (Colberg et al. 1995) as well as health risk in adults (Robinson et al. 2015). After a 6 minute warmup at 10 %PPO, they will complete four 4 min stages at 20, 30, 40, and 50 %PPO on the stationary bike, during which they breathe into a three way valve and wear the headgear. Blood will be drawn from a fingertip (20 microliters, about the size of a pinhead) pre- and post-exercise as well as at 30 %PPO using a lancet and portable monitor. Standardized procedures for blood collection will be followed.

They will return at the same time of day at least 24 h later and complete day 1 of HIIT, consisting of 8 1 min bouts on the stationary bike at intensity = 130%VT interspersed with 1 min of recovery at 10 %PPO. Training sessions will take < 30 minutes. VT obtained from the initial VO2max test will be used to determine individual workloads for all subjects' training sessions. In addition, 3 categorical scales assessing participants' psychological responses to training, Rating of Perceived Exertion (RPE, Borg CR10), affect (+5 to -5, Hardy & Rejeski 1991), and Physical Activity Enjoyment (PACES) will be assessed on days 3, 6, and 9 of training. Training sessions 2-3 will mirror this exercise prescription; whereas, days 4-6 will require 9 bouts at 130 %VT, and days 7-9, 10 bouts at 130 %VT. During all sessions, HR will be assessed continuously using the HR monitor. Participants will be required to abstain from voluntary physical activity and maintain their dietary intake during the study, which will be verified with a written log obtained pre- and post-training. Forty eight hours after the final training session, participants will repeat the baseline assessment of VO2max, following the required pre-test guidelines. And at least 48 h after this, they will complete the post-training measure of FatOx and CHOOx following identical procedures.
In fall 2017, we implemented this identical protocol in 14 active adults, so my Lab is very familiar with this type of study.

B) Where will the research be conducted? Describe any risks or confidentiality issues related to using this location.

Research will be conducted in the Human Performance Lab at CSUSM, ACD 111-115. All testing will be done in a room with a closed door and partition, and be held outside of regular class time and other lab activities to preserve participant identity.

C) State the specific dates/timeframe in which you plan to conduct your research.
Data collection will start approximately 3-20-18 and ensue through fall 2018 to give ample time for the PI to recruit the desired number of subjects.

5. Participant Debriefing or Feedback.
If deception is involved in your research, participants should be debriefed about the nature of the study as soon as possible. Participants should be given the opportunity to request a copy of the results of the study/your final report.

A) Describe any feedback or information you will offer participants.
The participants will not be deceived during the course of this study; however their baseline data will not be divulged at that time, to not skew their responses to training. For example, all screens showing physiological data will be turned away from view of the subject. At study termination, the PI will explain to each participant the meaning and/or application of his/her data and how it can be used in proper exercise programming. When these results are published, the PI will make a copy of this available to all participants.

6. List risks for each population participating in the research and for each methodology. Please be sure the risks listed here match the risks mentioned in your consent letter or information sheets.

Consider all risks very carefully. For more information on risks, see Examples of Risk.
A) Explain potential risks to your participants. Risks may be physical, psychological (e.g., strong emotional reactions to research questions), or inconveniences (e.g., time required).

1. There is potential for soreness and/or fatigue resulting from the exercise bouts, as well as potential for nausea and/or dizziness as well as small onset of injury. We recognize that prospective subjects are sedentary, yet hundreds of studies show that HIIT is well tolerated even in persons who are inactive, so these risks are relatively minimal, especially since these men and women are healthy and non-obese.
2. During VO2max testing, there is a small risk of cardiac event. In addition, men and women may feel a little uncomfortable taking their shirts off (men) or wearing a sports bra (women) during the VO2max test.
3. Potential for coercion to participate in the study.
4. Potential loss of time, financial gain, or employment due to the time demands of the study.
5. Potential for your identity to be revealed through participation in the study.
6. Potential for breach of confidentiality of data.
7. Chance of slight pain or bruising to be experienced during the finger stick blood samples.
Please note that use of the heart rate strap and facemask and breathing valve is not considered risks but are inconveniences which are characteristic of exercise testing. Hence, they are not denoted in the above list. Moreover, all equipment is sanitized using widely-accepted procedures (rinse with soap, water, and bleach) so there is no risk of contamination either.

Pregnant women, human fetuses, neonates (see Federal Guidelines, 45CFR26, subpart B)
Prisoners (see Federal Guidelines, 45CFR26, subpart C)
Children (see Federal Guidelines, 45CFR26, subpart D)
Other Vulnerable Populations such as persons with cognitive disabilities, economically or educationally disadvantaged
persons, etc.
C) Describe and special risks to vulnerable populations or your population profile
N/A

D) List risks related to confidentiality of data. What could happen if an unauthorized person accessed the data? For instance, participants' identify or personal information could be known by others
There is always a risk that participants' data could be accessed by persons other than the PI. In the case that it is accessed by unauthorized personnel, the data itself reveals nothing extensively personal about the participant, and the data will not be fully understood by persons not affiliated with the study.

E) Will any personal identifying data be recorded? If so, what information will be recorded? (e.g., Social security number, drivers license number, student id, address, phone number, birth date, personal email address)
A health history questionnaire will be completed by participants at baseline which will include contact information such as phone number and email address as means of contact between participants and the PI and his team. However, this will be placed in a separate area than the folders containing all participant data.

Safeguard Procedures to Minimize Risks.
A) Please respond to each risk that you listed in #6 above. State how you will minimize each risk and protect confidentiality.
1. Soreness, leg pain, nausea, and/or fatigue will be minimized by requiring subjects to complete a cool-down after each bout of exercise, as previously used (Burgomaster et al., 2006; 2007). Moreover, these men and women are non-obese and healthy so any incidence of this will be brief and likely of minimal magnitude. Similar procedures to these were used in many recent studies in persons performing HIIT in my Lab without incident (Astorino et al. 2017a, 2017b; Astorino et al., 2018; Thum et al, 2017; Wood et al. 2016, etc.), so chance of injury is unexpected.
2. Potential for cardiac events during the VO2max test will be minimized by using the health-history questionnaire to screen subjects for possible increased risk, which would exclude them from participating in the study. Risk of cardiac events in older inactive persons with elevated health risk is approximately 1 in 20,000, so the risk in young people is miniscule. And unfortunately, there is no alternative to the measurement of cardiac performance using the 6 electrodes, which is considered a non-invasive test. Only members of the research team will be in the Lab when this is being done.
3. Subjects will not be coerced in any way to partake in the study, and they will be reminded that they can withdraw from the study at any time without consequences, and only the Primary Investigator will be aware of their choice to stop their participation.
4. Participation is approximately 2 hours per week over 3 weeks, so this is not excess demand on participants' time. Moreover, researchers will do their best to make all visits to the Lab as

smooth and time-efficient as possible. And the financial incentive is there to somewhat compensate these men and women for their time.
5-6. Participants' names on all documents (data sheets, etc.) will be replaced by a code developed by the Primary Investigator to conceal their identity. Data-containing folders will be placed in locked cabinets only accessible to the PI.
7. Any pain experienced during the fingerstick procedure is brief, and the volume of blood drawn is extremely minute to have no impact on subjects' health status.

B) How you will safeguard data? Where/how will data be stored? Who will have access to the data? How will access be limited?
All data will be coded using participants' initials and subject number, so their name will not be linked at any point with their data file. Their data will be maintained in locked cabinets and will only be accessed by the Primary Investigator, who is the only person who will analyze data to publish manuscripts in peer-reviewed journals. Other personnel will not have access to these data. Data will be maintained by the Primary Investigator for at least three years after the completion of the project. Findings disseminated at conferences or in publications will be expressed in aggregate.

C) List referrals and/or resources that may be offered if a participant has a strong emotional response or a physical injury (e.g., clinics or shelters, medical or psychological referrals).
All subjects will be referred to their personal physician or the Student Health Center in the event of injury.

8.Study Benefits
A) Discuss any potential individual and/or societal benefits. Note, often there is no direct benefit for the participants. However, the study may contribute to the literature and/or future research.
Participants will benefit from the testing which will give them a measure of aerobic fitness and fat use, which are pertinent to the health of all persons. It is likely that HIIT will elicit gains in fitness and health status, which are extremely beneficial. However, the investigators cannot guarantee that these benefits will be accrued by all participants. In addition, these data will add to the body of knowledge concerning various responses to HIIT.

9. Researcher(s) qualifications and experience.
A) Briefly outline the primary researcher(s)'s qualifications and experiences relative to the subject of this research.
The Primary Investigator is a board-certified, doctorally-trained Exercise Physiologist expert in cardiorespiratory testing/exercise physiology, substrate metabolism, and sports nutrition. He has participated in research involving human performance testing throughout the preceding 18 years. His role in research has primarily been as lead investigator, giving him the ultimate responsibility to design proper research procedures, collect and analyze data, and disseminate data through professional presentations and manuscript writing. He first completed a HIIT study in 2009-2010 that resulted in 3 publications in peer-reviewed journals and multiple invited presentations and abstracts at scientific meetings, and a follow-up study completed in January 2013 led to 4 additional publications in top-tier journals. In May 2016, he finished a 28 mo

study leading to 3 publications in scientific journals as well as an invited tutorial at the recent ACSM meeting in Denver, CO in 2017 and annual SWACSM meeting in fall 2017. Last fall, he completed a HIIT study using this identical regime in active men and women. This extensive experience combined with his success mentoring undergraduate and graduate students in research provides him the foundation to initiate and eventually complete this study leading to publication in high-quality journals.

C) If using student or research assistants, please state how you will ensure that these assistants are trained and qualified to assist. All assistants should complete the CITI training on the protection of human participants in research.

All student assistants (at this time, TBD) who have at minimum BS degrees in Kinesiology will be exhaustively trained by the PI and have prior research experience. They will also complete CITI training.
